# Lipid Body–Phagosome Interaction in Macrophages during Infectious Diseases: Host Defense or Pathogen Survival Strategy?

**DOI:** 10.1371/journal.ppat.1002729

**Published:** 2012-07-05

**Authors:** Rossana C. N. Melo, Ann M. Dvorak

**Affiliations:** 1 Laboratory of Cellular Biology, Department of Biology, Federal University of Juiz de Fora (UFJF), Juiz de Fora, Minas Gerais, Brazil; 2 Department of Pathology, Beth Israel Deaconess Medical Center, Harvard Medical School, Boston, Massachusetts, United States of America; International Centre for Genetic Engineering and Biotechnology, India

## Abstract

Phagocytosis of invading microorganisms by specialized cells such as macrophages and neutrophils is a key component of the innate immune response. These cells capture and engulf pathogens and subsequently destroy them in intracellular vacuoles—the phagosomes. Pathogen phagocytosis and progression and maturation of pathogen-containing phagosomes, a crucial event to acquire microbicidal features, occurs in parallel with accentuated formation of lipid-rich organelles, termed lipid bodies (LBs), or lipid droplets. Experimental and clinical infections with different pathogens such as bacteria, parasites, and viruses induce LB accumulation in cells from the immune system. Within these cells, LBs synthesize and store inflammatory mediators and are considered structural markers of inflammation. In addition to LB accumulation, interaction of these organelles with pathogen-containing phagosomes has increasingly been recognized in response to infections and may have implications in the outcome or survival of the microorganism within host cells. In this review, we summarize our current knowledge on the LB-phagosome interaction within cells from the immune system, with emphasis on macrophages, and discuss the functional meaning of this event during infectious diseases.

## Introduction

**Figure 1:**
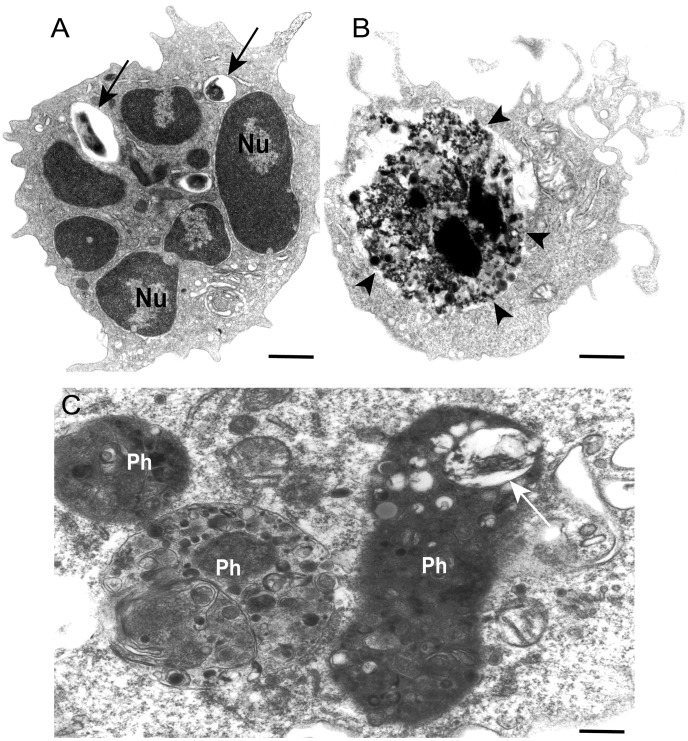
Ultrastructure of nascent phagosomes and phagolysosomes within phagocytic cells in a murine model of tuberculosis. (A) Nascent phagosomes containing phagocytosed mycobacteria (arrows) are seen in the cytoplasm of a neutrophil. In (B), macrophage pseudopods encircle an apoptotic cell (arrowheads). (C) Typical phagolysosomes (Ph) within a macrophage show heterogenous content and varying sizes and electron-density. A degenerating bacterium is observed (arrow). Mice were infected with *Mycobacterium bovis* bacillus Calmette-Guerin (BCG) and cells from the pleural cavity processed for transmission electron microscopy as before [3]. Scale bars, 1 µm (A, B), 500 nm (C).

Phagocytosis of invading microorganisms by specialized cells such as macrophages and neutrophils is a key component of the immune innate response. Phagocytosis is also a fundamental process for removal of cells undergoing apoptosis. The first stage of the elimination process is the internalization of the pathogens or apoptotic bodies into a plasma membrane-derived vacuole, known as phagosome (Figure 1A and 1B). Newly formed phagosomes, however, lack the ability to kill pathogens or to degrade the ingested targets. These properties are acquired during the course of phagosome maturation when the phagosome membrane and contents undergo considerable remodeling to transform the initially inert environment into a microbicidal one. Phagosomes mature by sequential fusion with endocytic (early and late endosomes) and lysosomal compartments culminating with the formation of the phagolysosome (Figure 1C), a highly acidic (pH between 4.0 and 5.0) compartment, in which the ingested pathogen is degraded (reviewed in [1], [2]).

In cells from the immune system, mainly macrophages, both pathogen phagocytosis and progression of pathogen-containing phagosomes generally occurs in parallel with accentuated formation of lipid-rich organelles, termed lipid bodies (LBs) or lipid droplets [3]–[7]. These organelles, largely associated with lipid storage in the past, are now recognized as dynamic and functionally active organelles, involved in a variety of functions such as lipid metabolism, trafficking, and signaling. LBs have also attracted considerable attention due to their link with human diseases such as obesity, inflammatory diseases, and cancer (reviewed in [8]–[10]). Experimental and clinical infections with different pathogens such as bacteria [3], [4], [11]–[15], parasites [5]–[7], [16], [17], and viruses [18], [19] induce LB accumulation within different cell types. One intriguing aspect of LBs formed in response to infections is the ability of these organelles to relocate in the cytoplasm and interact with phagosomes, suggesting a significant and yet ill-understood association between these structures [3]–[6], [12], [14]. This interaction occasioned attention because it may have implications for the microorganism outcome or survival within host cells. Here, we summarize our current knowledge on the LB-phagosome interaction within cells from the immune system, with emphasis on macrophages, key players in the initial resistance to the infection, and discuss the functional meaning of this event during infectious diseases.

## LB Structure and Composition

**Figure 2:**
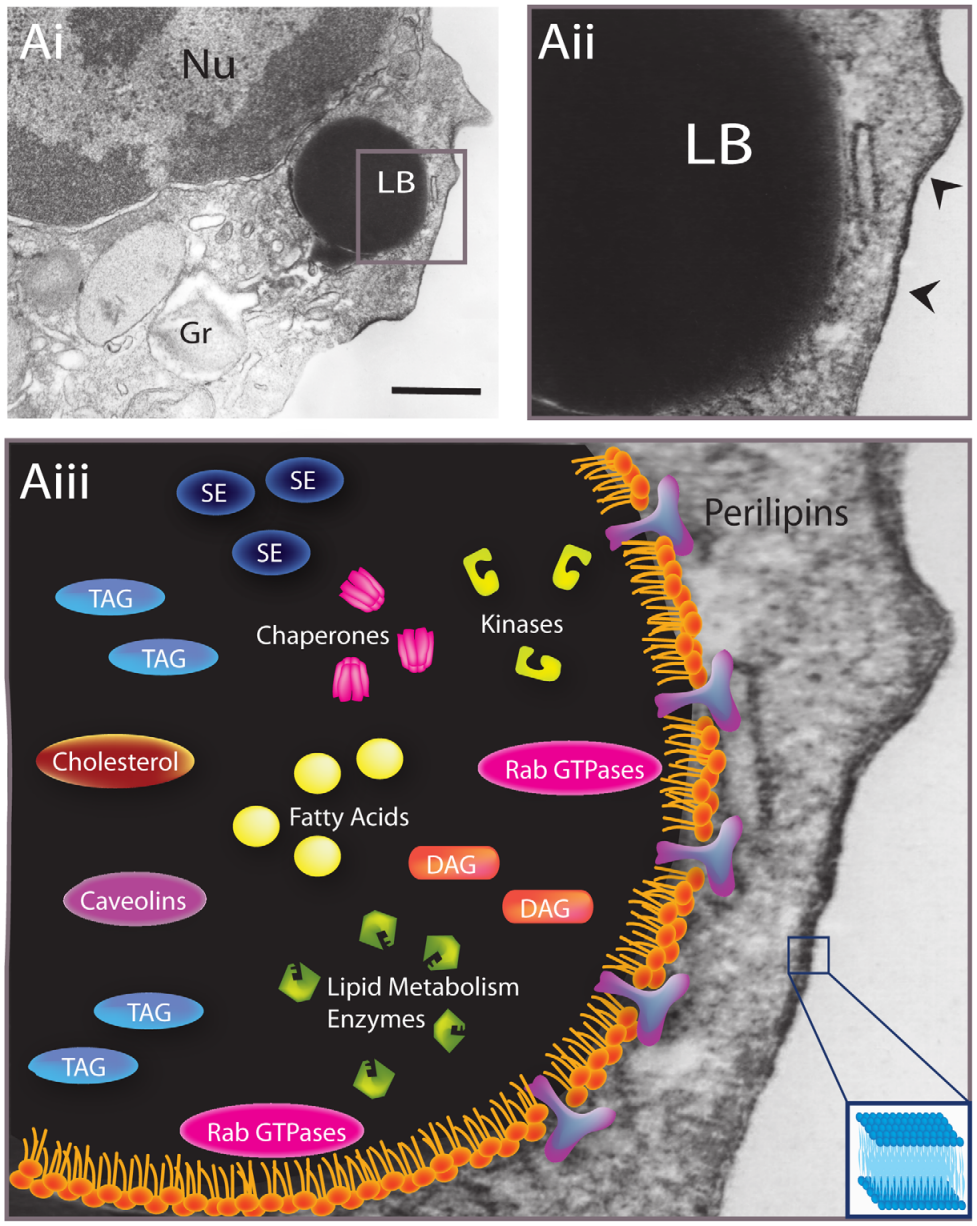
Lipid body (LB) structure and composition. (Ai–Aiii) A LB within a human blood eosinophil is observed by transmission electron microscopy (TEM) at different magnifications (boxed area in Ai is shown in Aii and Aiii). LBs are delimited by a monolayer of phospholipids differing from the structural organization (phospholipid bilayer membrane) of all other organelles, vesicles, and plasma membrane (arrowheads in Aii and box in Aiii). Structural proteins from the perilipin family are associated with the LB surface while the LB core contains mainly sterol esters (SE), triacylglycerols (TAG), diacilglycerols (DAG), and cholesterol. Numerous proteins are frequently found in LBs such as Rab GTPases, lipid metabolism enzymes, kinases, caveolins, and chaperones. Nu, nucleus; Gr, secretory granule. Scale bar, 600 nm.

LBs are intracellular organelles of all cell types including plants and microorganisms (reviewed in [20]). Despite variations in function, appearance, and composition between different organisms and their cell types, all LBs are recognized by a distinctive architecture—the presence of a core containing neutral lipids mainly tryacylglycerols (TAG) and sterol esters (SE) surrounded by a phospholipid hemimembrane with associated proteins [20], [21]. Therefore, in contrast to all cytoplasmic organelles and vesicles that have an aqueous content surrounded by a phospholipid bilayer membrane, the LB surface lacks a delimiting unit membrane structure (Figure 2). This unique feature of LBs facilitates the identification of these organelles by transmission electron microscopy (TEM) compared to other intracellular membranous organelles (Figure 2) [6].

LBs contain a collection of proteins with numerous biological functions. LB-specific structural proteins, the PAT family of proteins (recently renamed to perilipin family proteins [22])—perilipin/PLIN1, adipose differentiation-related protein (ADRP/Adipophilin/PLIN2) [23], and tail-interacting protein of 47 kDa (TIP47/PLIN3) [24]—are constitutively associated with the circumferential rim of LBs (Figure 2) and participate in the regulation of cellular lipid metabolism [25]. Moreover, enzymes of lipid metabolism, membrane trafficking proteins including small GTPases of the Rab family (critical regulators of vesicular traffic and organelle interaction), endoplasmic reticulum (ER) proteins, and molecular chaperones are frequently identified in LB fractions (Figure 2) [26]. In fact, a growing list of proteins, provided mainly by proteomic studies, has been described in association with LBs as summarized in several reviews [27]–[29]. The LB protein composition greatly varies depending on the cell type and its physiological state. Interestingly, LBs may act as platforms for managing the availability of proteins, functioning as transient sites for proteins that will be released, delivered, or destructed [30]. However, how proteins are specifically targeted to LBs is still poorly understood.

It has been recognized that proteins are not restricted to the LB surface, but they are also embedded in the LB core. For example, freeze-fracture immunocytochemistry and EM revealed that perilipin, caveolin-1, ADRP, and TIP47 are present in the LB cores of adipocytes and macrophages [31]. How polar proteins such as TIP47 and ADRP are arranged within these organelles remains to be defined.

### LB Composition in Cells from the Immune System

As noted, LBs within a cell differ regarding function and metabolic status and these characteristics are reflected in their composition. In cells from the immune system, LBs are recognized as sites for generation of inflammatory mediators (eicosanoids) and therefore have specific molecules linked to this synthesis (reviewed in [8], [32]).

LBs from macrophages, eosinophils, neutrophils, and mast cells contain stores of arachidonic acid (AA) associated with pools of phospholipid and/or neutral lipids [33]–[35]. AA is a 20-carbon fatty acid and a key signaling molecule acting as intracellular second messenger, as paracrine mediator of cell activation, and as a substrate for enzymatic conversion into eicosanoids [36].

The major enzymes involved in the enzymatic conversion of AA into eicosanoids are also present within LBs from activated cells of the immune system, mainly macrophages, eosinophils, and neutrophils. These enzymes include cyclooxygenases (COX) [3], [7], [33], [37]–[39], 5-and 15-lypoxygenases (5-LO and 15-LO) [39], [40], and leukotriene C_4_ (LTC_4_)-synthase [39]. Moreover, phospholipase A_2_ (cPLA_2_) and its activating protein kinases, mitogen-activated protein (MAP) kinases (ERk1, ERK2, p85, and p38), the upstream involved in AA liberation, have been described within LBs [41].

Overall, LBs from cells of the immune system compartmentalize the substrate (AA) and the entire enzymatic machinery for eicosanoid synthesis. Because eicoisanoids are non-storable mediators, newly formed and rapidly released upon cell stimulation, the detection of these molecules is not simple. However, by means of a new strategy to cross-link newly formed eicosanoids at its sites of synthesis, the presence of eicosanoids has been directly demonstrated within LBs [42]. prostaglandin E_2_ (PGE_2_) was found in LBs from mouse macrophages infected with *Mycobacterium bovis BCG*, which causes bovine tuberculosis [3], or with the intracellular protozoan parasite *Trypanosoma cruzi*, the causal agent of Chagas' disease, a debilitating cardiac illness [7]; LTC_4_ was demonstrated in LBs from human eosinophils and basophils stimulated with the chemokines eotaxin/CCL11 and RANTES/CCL5 [43] and in eosinophil LBs from murine models of allergic inflammation [44], and LTB_4_in LBs from neutrophils and macrophages during sepsis [45].

In summary, there is good evidence that LBs are able to change their composition in concert with cell activation acting as inflammatory organelles with roles in the innate immune response to infections and inflammatory processes.

## Pathogen Induction of LB Formation

Pathogens induce several changes in the host cell signaling and trafficking mechanisms. One prominent pathogen-mediated change is the formation of LBs in the host cell cytoplasm. Most cells contain a small number of cytoplasmic LBs, but they can be rapidly stimulated to form new LBs under interaction with pathogens. This interaction is also able to increase LB si*z*e and to induce LB ultrastructural alterations. LB biogenesis is a process that happens in vivo and in vitro in response to a variety of pathogens.

### Parasites

**Figure 3:**
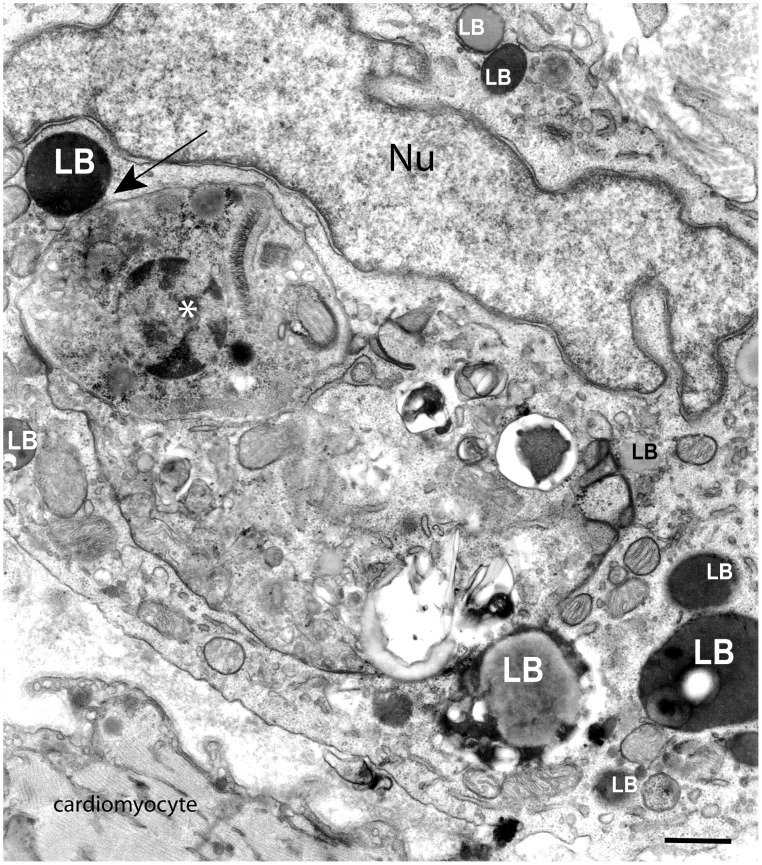
Lipid bodies (LBs) increase in number and interact with phagosomes within heart inflammatory macrophages during parasite infection. LBs with different sizes are seen as electron-dense or electron-lucent organelles surrounding and in contact (arrow) with a large phagolysosome containing an intact amastigote (*), the intracellular form of the parasite *Trypanosoma cruzi*. Rats were infected with the Y strain of *T. cruzi* and samples of the heart, a target organ of the parasite, processed for transmission electron microscopy at day 12 of infection [6], [68], [69]. Nu, nucleus. Scale bar, 800 nm.

The first observation of newly formed LBs in response to an in vivo parasite infectious disease dates to 2003 [5]. By investigating inflammatory macrophages from rats infected with a virulent strain of *T. cruzi* (Y strain), a significant increase of the LB numbers in peritoneal macrophages at day 6 and 12 of the infection was found. While control peritoneal macrophages presented ∼2.19±0.4 (mean ± SEM) LBs/cell, peritoneal macrophages from infected animals showed ∼18.09±1.4 LBs/cell at day 12 of infection. At this time, the most intense inflammatory process and parasitism in the heart, a target organ of Chagas' disease, compared to other points during the acute phase in rats, is observed [5]. Accordingly, inflammatory macrophages recruited to the heart exhibited a striking increase in LB numbers (Figure 3) [5]. *T. cruzi* is also capable to induce in vitro LB formation within mouse peritoneal macrophages through a Toll-like receptor -2 (TLR2)-dependent mechanism [7]. At 24 h of murine infection, both the cells containing internalized parasites as well non-parasitized cells showed an increased number of LBs compared to control, non-infected cells, suggesting a bystander amplification of the response [7]. Interestingly, parasitized cells showed a significantly higher number of LBs (3-fold) compared to non-parasitized cells, demonstrating that the uptake of the parasite directly induces LB biogenesis [7].

Pathological studies of target organs of malaria, such as kidney and liver, found the presence of a high number of LBs in infected mice, indicating that the parasite *Plasmodium berghei*, the causative agent of the disease, induces LB accumulation in host cells [46], [47]. Other parasites such as *Toxoplasma gondii*, which causes human toxoplasmosis [16], and *Leishmania amazonensis*, a causal agent of human leishmaniasis [48], trigger LB formation during the in vitro infection of human fibroblasts and mouse peritoneal macrophages, respectively.

### Bacteria

Interaction of pathogenic bacteria with host cells leads to LB biogenesis. Tuberculosis caused by *Mycobacterium tuberculosis* is characterized by a tight interplay between *M. tuberculosis* and host cells within cellular aggregates (granulomas) [4], [49]. The induction of foamy macrophages—a granuloma-specific cell population characterized by its high lipid content compartmentalized in LBs—has been extensively reported during the progression of tuberculosis caused by *M. tuberculosis* in both humans and experimental settings [4], [13], [49]. In experimental studies with *Mycobacterium bovis* bacillus Calmette-Guerin (BCG), it was found that this pathogen is capable of inducing a dose- and time-dependent increase on LB formation within pleural macrophages [3]. LB formation initiates rapidly and significantly increased LB numbers are noted within 1 h, reaching maximum levels within 24 h, and remaining increased for at least 15 d after BCG infection [3]. Remarkably, nonsteroidal anti-inflammatory drugs (NSADs), such as aspirin and NS-398, drastically inhibited *M. bovis BCG*-induced LB formation within 24 h. In parallel, the BCG-induced PGE_2_ generation was completely abrogated [3].

Accumulation of lipid-filled foamy macrophages is also a hallmark of lepromatous leprosy, a chronic disease caused by *Mycobacterium leprae*. Leprosy is characterized by widespread skin lesions in which *M. leprae* lives and replicates in foamy macrophages. These macrophages are highly positive for ADRP [11], [50] and perilipin [50]. Moreover, *M. leprae* infection increases expression of ADRP/perilipin mRNA in THP-1 cells, a human promonocytic cell line [50]. These observations indicate that the foamy aspect of macrophages is derived from LB accumulation induced during *M. leprae* infection. In fact, the capacity of *M. leprae* to induce LB formation was confirmed in vivo via an experimental model of mouse pleurisy and in in vitro studies with human monocytes and murine peritoneal macrophages [11].

LB formation within macrophages is also driven by infection with *Chlamydia pneumonia* and characterizes the early atherosclerosis in the presence of low-density lipoprotein (LDL) [15]. More recently, an in vivo ultrastructural study during the initial infection with *Chlamydia muridarum,* which causes genital infection in mice, demonstrated that epithelial host cells accumulate LBs in parallel to bacteria replication [51].

Interestingly, the bacterium uptake does not seem essential for LB formation within macrophages and other cells. In human peripheral blood monocytes and murine macrophages exposed to *M. leprae*, LB biogenesis is observed in the cytoplasm of both cells bearing bacteria and cells with no bacteria [11]. However, as noted in macrophages cultured with T. *cruzi* [7], cells with internalized pathogens showed higher LB formation compared to cells that were exposed to the pathogens but did not engulf them [11], indicating that phagocytosis potentiates LB biogenesis.

Bacterial derivates such as lipopolysaccharide (LPS) present in all Gram-negative bacteria [15], [52] and the mycobacterial cell wall component lipoarabinomannan (LAM), a virulence factor of *M. tuberculosis* [3], are also able to induce LB formation in macrophages.

Distinct signaling pathways can trigger LB formation within cells from the immune system. Specific bacteria- and receptor-mediated pathways activate intracellular signaling that leads to enhanced LB formation. For instance, *M. bovis* BCG [3], but not the non-pathogenic bacteria *Mycobacterium smegmatis* or *Bacillus subtilis* [3], induces toll-like receptor 2 (TLR2)-mediated formation of LBs in macrophages; TLR6 but not TLR2 drives LB biogenesis in *M. leprae*-infected Schwann cells [53], and TLR2 but not TLR4 are involved in the formation of LBs in macrophages infected with *Chlamydia pneumonia* [15].

### Viruses

LB formation is also induced by infection with viruses such as the hepatitis C virus (HCV), the major causative pathogen associated with liver cirrhosis and hepatocellular carcinoma [18], and dengue virus (DENV), an emerging viral disease transmitted by arthropods to humans in tropical countries [19]. Pharmacological inhibition of LB formation greatly decreases DENV and HCV replication [19], [54], suggesting LBs as targets for antiviral strategies.

A list of pathogens that induce LB biogenesis within different mammalian cells is shown in Table 1.

**Table 1:** Pathogen-induced lipid body (LB) formation and LB-phagosome interaction in mammalian cells.

Pathogen	LB Formation	LB-Phagosome Interaction	Cell Type	Organism	Refs
**Bacteria**					
*Acinetobacter baumannii*	+	n.d.	J774 macrophages	Mouse	[70]
*Bacillus subtilis*	−	−	Macrophages	Mouse	[3]
*Chlamydia muridarum*	+	+	Epithelial cells (cervix)	Mouse	[51]
*Chlamydia pneumoniae*	+	n.d.	Macrophages	Mouse	[15]
*Chlamydia trachomatis*	+	+	Hep2 cell line	Human	[56]
	+	+	HeLa cell line	Human	[14]
*Escherichia coli*	n.d.	+	THP-1 cell line (macrophage-like)	Human	[61]
	+	n.d.	J774 macrophages	Mouse	[70]
*Klebsiella pneumonia*	+	n.d.	Peripheral blood monocytes	Human	[70]
	+	n.d.	J774 macrophages	Mouse	[70]
*Mycobacterium bovis* BCG	+	+	Pleural macrophages	Mouse	[3]
*Mycobacterium leprae*	+	n.d.	Skin macrophages	Human	[11]
	+	n.d.	Peripheral blood monocytes	Human	[11]
	+	n.d.	Peritoneal macrophages	Mouse	[11]
	+	n.d.	Pleural macrophages	Mouse	[11]
	+	+	Schwann cells	Human	[12]
*Mycobacterium smegmatis*	−	−	Macrophages	Mouse	[3]
*Mycobacterium tuberculosis*	+	+	Foamy macrophages (granuloma)	Human	[4]
	+	n.d.	Macrophages	Human	[13]
*Proteus vulgaris*	+	n.d.	J774 macrophages	Mouse	[70]
*Pseudomonas aeruginosa*	+	n.d.	J774 macrophages	Mouse	[70]
*Pseudomonas diminuta*	+	n.d.	J774 macrophages	Mouse	[70]
*Staphylococcus aureus*	+	n.d.	J774 macrophages	Mouse	[70]
*Staphylococcus epidermidis*	+	n.d.	J774 macrophages	Mouse	[70]
*Staphylococcus salivarius*	+	n.d.	Peripheral blood monocytes	Human	[70]
	+	n.d.	J774 macrophages	Mouse	[70]
*Vibrio cholera*	+	n.d.	Mucosal mast cells	Human	[71]
**Bacteria Derivates**					
CpG-DNA	+	n.d.	J774 macrophages	Mouse	[70]
Flagellin	+	n.d.	J774 macrophages	Mouse	[70]
LPS (lipopolysaccharide)	+	n.d.	Macrophages	Mouse	[15], [52], [70]
LAM (lipoarabinomannan)	+	n.d.	Macrophages	Mouse	[3]
**Parasites**					
*Leishmania amazonensis*	+	n.d.	Peritoneal macrophages	Mouse	[48]
*Plasmodium berghei*	+	n.d.	Kidney cells	Mouse	[47]
	+	n.d.	Liver cells	Mouse	[46]
*Schistosoma mansoni* derivates	+	n.d.	Eosinophils	Mouse	[17]
*Toxoplasma gondii*	+	n.d.	Fibroblasts	Human	[16]
*Trypanosoma cruzi*	+	+	Heart macrophages	Rat	[5], [6]
	+	+	Peritoneal macrophages	Rat	[5], [6]
	+	n.d.	Peritoneal macrophages	Mouse	[7]
**Viruses**					
*Dengue virus*	+	n.a.	BHK-21 cells	Hamster	[19]
*Hepatitis C virus*	+	n.a.	CHO and HepG2 cell lines	Human	[18]
**Fungi**					
*Candida albicans* derivates	+	n.d.	Macrophages	Rat	[72]
	+	n.d.	Hepatocytes	Rat	[72]

+, induced; −, not induced; n.d., not determined; n.a., not applicable.

### Pathogen-Mediated LB Structural Changes

Newly formed LBs within pathogen-infected macrophages can show changes in si*z*e and ultrastructure. In scoring the diameters of LBs within inflammatory macrophages from rats experimentally infected with *T. cruzi*, 74% of LBs had a size <0.5 µm in non-infected whereas 54% of LBs from infected animals were >0.5 µm, reaching up to 3 µm. When macrophages from *T. cruzi*-infected animals were challenged in vivo with higher parasite load, a significant increase of LB sizes compared to LBs induced by the infection alone was observed [6].

One interesting ultrastructural aspect of LBs is their electron-density (osmiophilia), which is dependent on the cell type and can change in response to pathogens. When observed by TEM, macrophages from mice infected with *M. bovis* BCG show distinct morphology, becoming larger and less dense organelles compared to LBs from non-infected cells [3]. Accordingly, LBs formed in response to the in vivo *T. cruzi* infection within inflammatory macrophages also exhibited changes in electron-density compared to uninfected cells [6]. Interestingly, LBs change their electron-density in macrophages stimulated in vivo with higher parasite load. Rats were exposed to a single, high dose of gamma irradiation 1 d before infection, which depletes the humoral and cellular immune responses except for the phagocytic activity of macrophages. Inflammatory macrophages from irradiated-infected animals showed an increase in the numbers of both light-dense and strongly electron-dense LBs compared to infection alone [6]. Pathogen-mediated LB morphological changes may reflect differences in lipid or protein composition, stages of formation of new LBs, mobilization, and/or neutral lipids/phospholipids ratio within LBs [6].

## Interaction of LBs with Phagosomes

**Figure 4:**
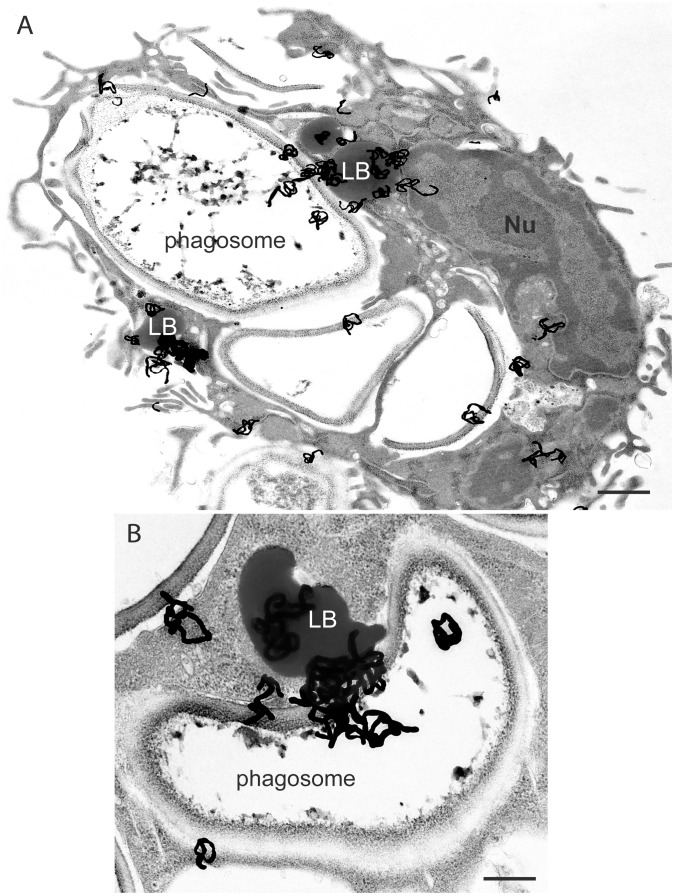
Human lipid bodies (LBs) observed with an ultrastructural method for autoradiography after a pulse of tritiated arachidonic acid and exposure to zymosan. (A) ^3^H-arachidonate incorporated by a human macrophage is localized predominantly in LBs in association with zymosan-filled phagosomes. In (B), a LB labeled with numerous silver grains is seen in higher magnification. Note that the labeled lipid content is projecting into the zymosan-containing phagosome lumen. Scale bars, 1 µm (A), 600 nm (B).

The first documentation of a significant interaction between LBs and phagosomes dates to 1983 [55]. In an autoradiographic ultrastructural study of the incorporation of ^3^H AA by macrophages that were also exposed to zymosan particles for phagocytosis, a striking approximation of ^3^H AA-incorporated LBs with phagolysosomes was observed (Figure 4). Moreover, ^3^H AA-labeled LBs occasionally fused with phagolysosomes membranes and many cells exhibited autoradiographic grains over phagolysosomes (Figure 4) [55]. This was the first evidence that LBs not only were able to associate with phagosomes but also discharged their contents into these structures [55]. However, this interaction received scant attention for two decades.

**Figure 5:**
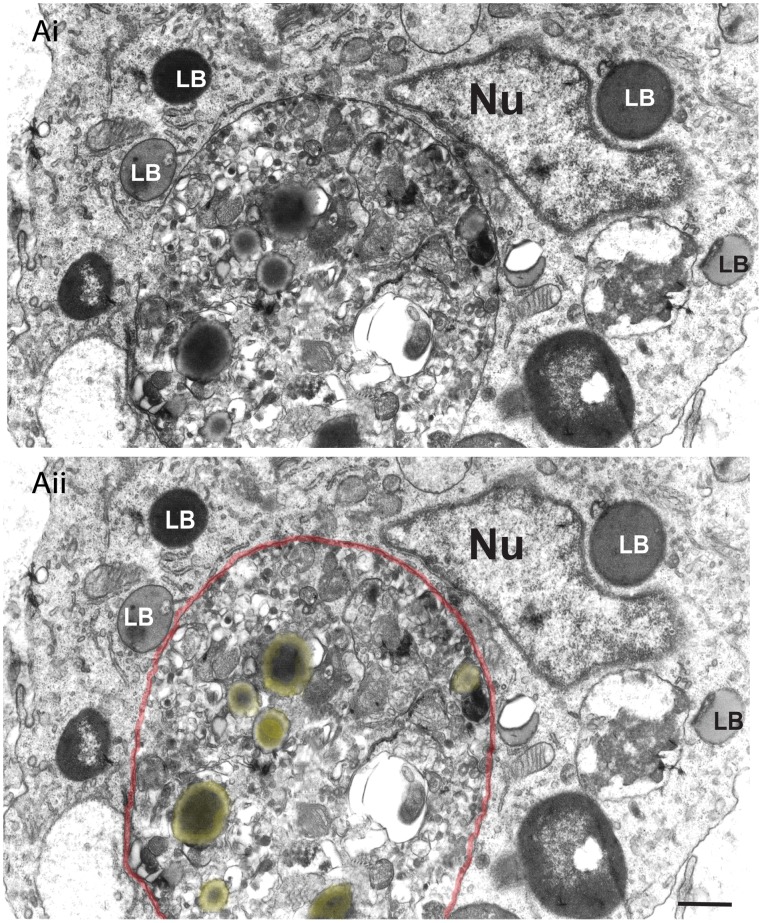
Lipid bodies (LBs) translocate to phagolysosomes in infected macrophages. (Ai) LBs with different electron-densities are observed around a large phagolysosome (outlined in red in Aii) in the macrophage cytoplasm. Note that several LBs (highlighted in yellow in Aii) are seen within the phagolysosome. Rats were infected with the Y strain of *T. cruzi* and heart samples processed for transmission electron microscopy at day 12 of infection [6], [68], [69]. Nu, nucleus. Scale bar, 600 nm.

In 2003, a study of inflammatory macrophages triggered by the in vivo infection with *T. cruzi* demonstrated a clear association of LBs with phagosomes in parallel to LB formation (Figures 3 and 5) [5]. Detailed quantitative TEM analyses of the LB-phagosome interaction induced by the experimental *T. cruzi* infection revealed that 47% of newly formed LBs were associated with phagolysosomes within inflammatory macrophages, mainly in the heart. LBs were seen surrounding or attached to phagosomes (Figures 3 and 5) and even within the lumen of these structures (Figure 5), indicating that the LB-phagosome interaction can result in LB internalization into parasite-containing phagosomes [6]. Later the identification by TEM of LB-phagosome interaction in macrophages from both animal models and humans infected with mycobacteria brought new attention to LBs as organelles connected with the life cycle of pathogens [3], [4].

Other studies have been demonstrating LB translocation into the lumen of bacteria-containing phagosomes using live cell fluorescence microscopy and/or TEM [12], [14], [51]. In an experimental model of infection with the intracellular pathogen *Chlamydia trachomatis*, which causes several ailments such as trachoma, conjunctivitis, epididymitis, and pelvic inflammatory disease, it was shown that LBs dock at the surface of the bacteria-containing vacuole (termed “inclusions”) within Hep2 or HeLa cells, penetrate the vacuole membrane, and intimately associate with reticulate bodies, the replicative form of *Chlamydia* [14], [56].

Nerve biopsies of patients infected with *M. leprae* also show accumulating LBs in close association with *M. leprae*-containing phagosomes within Schwann cells, a target cell of this pathogen. These LBs are then promptly recruited to the bacteria-containing phagosomes, a process that depends on cytoskeletal reorganization and phosphatidylinositol 3 kinase (PI3K) signaling [12]. Thus, the lipid-laden, bacterial-bearing vacuoles observed in heavily infected SCs in lepromatous leprosy nerve biopsies might be formed by the continuous formation and recruitment of LBs, giving rise to their foamy appearance [12]. Another in vivo study using a model of intracervical murine infection with *Chlamydia muridarum* demonstrated the same phenomenon—multiple LBs in contact with the pathogen-containing vacuole and LBs entering into the vacuole within host cells [51].

Overall, infection with different pathogens leads to movement of LBs into phagosomes. It is intriguing how pathogens target LB and how intact and non-membrane-bound organelles such as LBs are translocated across the phagosome membrane. Bacterial proteins seem to be involved in capturing LB into bacteria-containing vacuoles while the translocation process seems to involve displacement of the LB structural protein ADRP from the LB surface to the phagosome membrane, as observed during the in vitro infection with *Chlamydia trachomatis* [14]. Interestingly, both ADRP and perilipin, were also immunolocalized on the membranes of bacilli-containing phagosomes in macrophages from skin biopsy specimens from patients with lepromatous leprosy [50]. However, the mechanistic details underlying the LB-phagosome interaction require further investigations to be deciphered.

LB-phagosome association has also been observed in other situations. Contact sites between LBs and latex bead-containing phagosomes were identified by high resolution Raman microscopy in neutrophilic granulocytes [57] and by fluorescence microscopy in dendritic cells [58]. By using time-lapse fluorescence microscopy, it was pointed out that the LB-phagosome association within neutrophilic granulocytes seems transient, similar to “kiss-and-run” behavior displayed by endosomes involved in phagosome maturation [57]. LBs were also observed in close contact with nascent autophagosomes within normal rat kidney (NRK) cells [59], but the biology of LBs during autophagy is still not understood.

## LB-Phagosome Interaction: Functional Implications

As noted, the LB-phagosome interaction seems to be a general event found during infections with different pathogens in both humans and experimental models. Although little is known about the functional meaning of this interaction, it raises intriguing possibilities in light of the LB composition and functions.

Much interest has been focused on LBs as conduits for the transport of potential nutrients, especially neutral lipids, to the phagosome. The lipid content of LBs may, therefore, serve as a nutrient source for the pathogen enabling its survival within the cell [3], [4], [12], [14], [53]. The LB-phagosome interaction has been considered as a pathogen strategy for accessing host lipids during *M. tuberculosis* [4] and *M. leprae* [12] human infections and experimental infection with *Chlamydia trachomatis* [14]. Considering that *M. tuberculosis* bacilli are able to accumulate lipids during dormancy from which it derives both carbon and energy for its own metabolism [60], the mycobacteria-phagosome interaction could be important for the pathogen growth and persistence [3], [4].

Pharmacological inhibition of LB formation within pathogen-infected cells was investigated. Using triacsin C, which prevents LD biogenesis by specifically inhibiting the activity of a subset of long chain acyl-coA synthetases (ACSL) required for triacylglyceride and cholesterol ester biosynthesis, a decrease was observed in the phagosome size and reduction of chlamydial growth within Hep2 cells [56]. The use of another inhibitor of lipid metabolism, C75, which inhibits fatty acid synthase (FAS), inhibited not only the *M. tuberculosis*-induced LB formation but also the bacterial viability in Schwann cells [53].

The possibility of LBs to deliver other nutrients into the phagosome for pathogen growth was also raised. Complexes of iron and mycobactins, lipophilic siderophores of mycobacteria, accumulate in LBs within macrophages infected with *Escherichia coli* [61]. It is suggested that a subsequent migration of iron-mycobactin complex from LBs to phagosomes would facilitate iron delivery to phagosomal mycobacteria, acting as an iron source for the pathogen and consequently promoting their growth [61].

Taken together, a picture emerges in which pathogens usurp host, newly-formed LBs to obtain nutrients, mainly lipids, as a survival strategy. Moreover, the enhanced capacity of host cells to generate inflammatory mediators in the course of pathogenic infections due to increased LB formation and compartmentalization of signaling and eicosanoid production within LBs may also be contributing to mechanisms that intracellular pathogens have developed to survive in host cells. For example, high concentration of PGE_2_ in macrophages act as a potent inhibitor of Th1 type response [62] and of nitric oxide (NO) production [63], creating thus an appropriate environment for optimal pathogen growth [63].

On the other hand, lipids have been gaining attention as co-directors of phagocytosis (reviewed in [64]). Lipids play multiple roles as determinants of phagosomal formation and fate and as coordinators of the recruitment and retention of key phagocytic proteins [64]. In *M. tuberculosis*-infected macrophages, selected lipids, including AA, can activate actin assembly, phagosome-lysosome fusion, and phagosome maturation, resulting in bacteria killing [65]. Lipids also help to activate the phagosome-resident enzyme nicotinamide adenine dinucleotide phosphate (NADPH)-oxidase [66], an event essential for the degradation of microbes upon infection [1]. Because several subunits of NADPH oxidase depend on AA, it was suggested that the AA content of LBs are used by phagocytes to locally activate NADPH-oxidase [57].

Key molecules, such as Rab 5 and Rab 7, are involved in the sequential interactions of early and late endosomes with phagosomes [1], [2]. Considering that LBs are sites for these GTPases (Figure 2) [26], [67], the association of LBs with phagosomes may constitute a mechanism for Rab transport to and from the phagosome for phagosome maturation [57]. Igtp (Irgm^3^), an ER-resident 47 KDa immune-related GTPase involved in phagosomal maturation and phagocytic cross-presentation, was also identified in LBs within dendritic cells, indicating that LBs regulate cross-presentation of phagocytosed antigens in these cells [58]. In addition, the presence of contact sites between LBs and phagosomes in dendritic cells may support a regulatory function of LBs on phagolysosomal progression [58]. Thus, the enigmatic LB-phagosome interaction cannot be solely viewed as a pathogen strategy to prolong and sustain its own survival, but also might be a host strategy to destroy or, at least, to “try” to kill the microbial invader.

## Summary and Perspectives

LBs emerge as key organelles involved in experimental and clinical infections with different pathogens, such as bacteria, parasites, and viruses. Within cells recruited in response to these infections, especially macrophages, LBs contribute to the genesis of inflammatory mediators and are considered as structural markers of inflammation. In addition to LB accumulation, interaction of these organelles with pathogen-containing phagosomes has increasingly been recognized. Recent observations have indicated that this intriguing and intimate association is a pathogen-driven process, evolved as a strategy to survive within the host cells by sequestering mainly host lipids. However, it should be noted that the LB-phagosome interaction may be linked to defense events in which the host cell seeks to kill the pathogen invader. Because phagosomal membrane and luminal contents must undergo remodeling to transform the initially inert environment into a microbicidal one, attention should be paid to LBs as potential determinant organelles in the phagocytic event. While this is more speculative, to date we cannot answer whether LBs have a major and a definitive role in the intracellular survival or destruction of pathogens and/or if these organelles are able to interfere with phagocytosis pathways. Other questions regarding how pathogens target LBs and/or how LBs target pathogen-containing phagosomes and are translocated into these vaccuoles await a lot of more investigations. Novel approaches such as electron tomographic analysis of the LB-phagosome interaction dynamics combined with further refined functional analyses will be required to address these questions. A better understanding of the cell biology of LBs and their potential role during pathogen phagocytosis may be crucial for the application of novel therapies addressing different pathological conditions.

**Figure 6:**
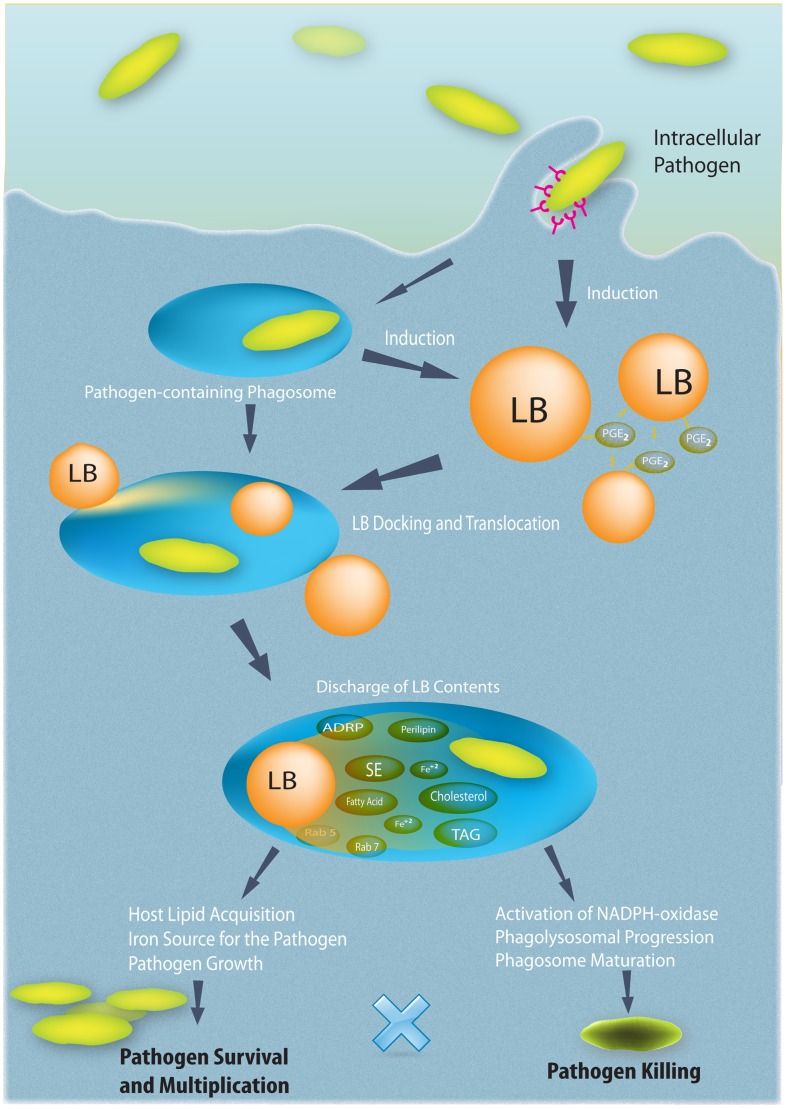
Schematic representation of pathogen-mediated LB formation, LB translocation to phagosomes, and possible consequences of the LB-phagosome interaction. Different pathogens induce LB formation within infected cells, such as macrophages. LB biogenesis occurs after both cell exposition to pathogens and/or receptor-mediated pathogen uptake. Cytoplasmic newly formed LBs relocate in the cytoplasm, dock at the surface of pathogen-containing phagosomes, and translocate into their lumen. This interaction may enable acquisition of nutrients for pathogen survival and multiplication or may be linked to host cell defense mechanisms favoring phagosome maturation and pathogen killing. Within macrophages and other inflammatory cells, LBs actively produce eicosanoids, such as prostaglandin E_2_ (PGE_2_), which may also contribute to pathogen outcome by inhibiting Th1 responses.

## References

[1] Flannagan RS, Cosio G, Grinstein S (2009). Antimicrobial mechanisms of phagocytes and bacterial evasion strategies.. Nat Rev Microbiol.

[2] Kinchen JM, Ravichandran KS (2008). Phagosome maturation: going through the acid test.. Nat Rev Mol Cell Biol.

[3] D'Avila H, Melo RCN, Parreira GG, Werneck-Barroso E, Castro-Faria-Neto HC (2006). Mycobacterium bovis bacillus Calmette-Guerin induces TLR2-mediated formation of lipid bodies: intracellular domains for eicosanoid synthesis in vivo.. J Immunol.

[4] Peyron P, Vaubourgeix J, Poquet Y, Levillain F, Botanch C (2008). Foamy macrophages from tuberculous patients' granulomas constitute a nutrient-rich reservoir for M. tuberculosis persistence.. PLoS Pathog.

[5] Melo RCN, D'Avila H, Fabrino DL, Almeida PE, Bozza PT (2003). Macrophage lipid body induction by Chagas disease in vivo: putative intracellular domains for eicosanoid formation during infection.. Tissue Cell.

[6] Melo RCN, Fabrino DL, Dias FF, Parreira GG (2006). Lipid bodies: structural markers of inflammatory macrophages in innate immunity.. Inflamm Res.

[7] D'Avila H, Freire-de-Lima CG, Roque NR, Teixeira L, Barja-Fidalgo C (2011). Host cell lipid bodies triggered by Trypanosoma cruzi infection and enhanced by the uptake of apoptotic cells are associated with prostaglandin E generation and increased parasite growth.. J Infect Dis.

[8] Bozza PT, Melo RCN, Bandeira-Melo C (2007). Leukocyte lipid bodies regulation and function: contribution to allergy and host defense.. Pharmacol Ther.

[9] Beller M, Thiel K, Thul PJ, Jackle H (2010). Lipid droplets: a dynamic organelle moves into focus.. FEBS Lett.

[10] Farese RV, Walther TC (2009). Lipid droplets finally get a little R-E-S-P-E-C-T.. Cell.

[11] Mattos KA, D'Avila H, Rodrigues LS, Oliveira VG, Sarno EN (2010). Lipid droplet formation in leprosy: Toll-like receptor-regulated organelles involved in eicosanoid formation and Mycobacterium leprae pathogenesis.. J Leukoc Biol.

[12] Mattos KA, Lara FA, Oliveira VG, Rodrigues LS, D'Avila H (2011). Modulation of lipid droplets by Mycobacterium leprae in Schwann cells: a putative mechanism for host lipid acquisition and bacterial survival in phagosomes.. Cell Microbiol.

[13] Daniel J, Maamar H, Deb C, Sirakova TD, Kolattukudy PE (2011). Mycobacterium tuberculosis uses host triacylglycerol to accumulate lipid droplets and acquires a dormancy-like phenotype in lipid-loaded macrophages.. PLoS Pathog.

[14] Cocchiaro JL, Kumar Y, Fischer ER, Hackstadt T, Valdivia RH (2008). Cytoplasmic lipid droplets are translocated into the lumen of the Chlamydia trachomatis parasitophorous vacuole.. Proc Natl Acad Sci U S A.

[15] Cao F, Castrillo A, Tontonoz P, Re F, Byrne GI (2007). Chlamydia pneumoniae–induced macrophage foam cell formation is mediated by Toll-like receptor 2.. Infect Immun.

[16] Charron AJ, Sibley LD (2002). Host cells: mobilizable lipid resources for the intracellular parasite Toxoplasma gondii.. J Cell Sci.

[17] Magalhaes KG, Almeida PE, Atella GC, Maya-Monteiro CM, Castro-Faria-Neto HC (2010). Schistosomal-derived lysophosphatidylcholine are involved in eosinophil activation and recruitment through Toll-like receptor-2-dependent mechanisms.. J Infect Dis.

[18] Barba G, Harper F, Harada T, Kohara M, Goulinet S (1997). Hepatitis C virus core protein shows a cytoplasmic localization and associates to cellular lipid storage droplets.. Proc Natl Acad Sci U S A.

[19] Samsa MM, Mondotte JA, Iglesias NG, Assuncao-Miranda I, Barbosa-Lima G (2009). Dengue virus capsid protein usurps lipid droplets for viral particle ormation.. PLoS Pathog.

[20] Murphy DJ (2001). The biogenesis and functions of lipid bodies in animals, plants and microorganisms.. Prog Lipid Res.

[21] Tauchi-Sato K, Ozeki S, Houjou T, Taguchi R, Fujimoto T (2002). The surface of lipid droplets is a phospholipid monolayer with a unique Fatty Acid composition.. J Biol Chem.

[22] Kimmel AR, Brasaemle DL, McAndrews-Hill M, Sztalryd C, Londos C (2010). Adoption of PERILIPIN as a unifying nomenclature for the mammalian PAT-family of intracellular lipid storage droplet proteins.. J Lipid Res.

[23] Brasaemle DL, Dolios G, Shapiro L, Wang R (2004). Proteomic analysis of proteins associated with lipid droplets of basal and lipolytically stimulated 3T3-L1 adipocytes.. J Biol Chem.

[24] Wolins NE, Rubin B, Brasaemle DL (2001). TIP47 associates with lipid droplets.. J Biol Chem.

[25] Bickel PE, Tansey JT, Welte MA (2009). PAT proteins, an ancient family of lipid droplet proteins that regulate cellular lipid stores.. Biochim Biophys Acta.

[26] Wan HC, Melo RCN, Jin Z, Dvorak AM, Weller PF (2007). Roles and origins of leukocyte lipid bodies: proteomic and ultrastructural studies.. FASEB J.

[27] Hodges BD, Wu CC (2010). Proteomic insights into an expanded cellular role for cytoplasmic lipid droplets.. J Lipid Res.

[28] Digel M, Ehehalt R, Fullekrug J (2010). Lipid droplets lighting up: insights from live microscopy.. FEBS Lett.

[29] Goodman JM (2009). Demonstrated and inferred metabolism associated with cytosolic lipid droplets.. J Lipid Res.

[30] Welte MA (2007). Proteins under new management: lipid droplets deliver.. Trends Cell Biol.

[31] Robenek H, Robenek MJ, Troyer D (2005). PAT family proteins pervade lipid droplet cores.. J Lipid Res.

[32] Melo RCN, D'Avila H, Wan HC, Bozza PT, Dvorak AM (2011). Lipid bodies in inflammatory cells: structure, function, and current imaging techniques.. J Histochem Cytochem.

[33] Dvorak AM, Weller PF, Harvey VS, Morgan ES, Dvorak HF (1993). Ultrastructural localization of prostaglandin endoperoxide synthase (cyclooxygenase) to isolated, purified fractions of guinea pig peritoneal macrophage and line 10 hepatocarcinoma cell lipid bodies.. Int Arch Allergy Immunol.

[34] Weller PF, Monahan-Earley RA, Dvorak HF, Dvorak AM (1991). Cytoplasmic lipid bodies of human eosinophils. Subcellular isolation and analysis of arachidonate incorporation.. Am J Pathol.

[35] Triggiani M, Oriente A, Seeds MC, Bass DA, Marone G (1995). Migration of human inflammatory cells into the lung results in the remodeling of arachidonic acid into a triglyceride pool.. J Exp Med.

[36] Yaqoob P (2003). Fatty acids as gatekeepers of immune cell regulation.. Trends Immunol.

[37] Dvorak AM, Morgan ES, Tzizik DM, Weller PF (1994). Prostaglandin endoperoxide synthase (cyclooxygenase): ultrastructural localization to nonmembrane-bound cytoplasmic lipid bodies in human eosinophils and 3T3 fibroblasts.. Int Arch Allergy Immunol.

[38] Dvorak AM, Morgan E, Schleimer RP, Ryeom SW, Lichtenstein LM (1992). Ultrastructural immunogold localization of prostaglandin endoperoxide synthase (cyclooxygenase) to non-membrane-bound cytoplasmic lipid bodies in human lung mast cells, alveolar macrophages, type II pneumocytes, and neutrophils.. J Histochem Cytochem.

[39] Bozza PT, Yu W, Penrose JF, Morgan ES, Dvorak AM (1997). Eosinophil lipid bodies: specific, inducible intracellular sites for enhanced eicosanoid formation.. J Exp Med.

[40] Bozza PT, Yu W, Cassara J, Weller PF (1998). Pathways for eosinophil lipid body induction: differing signal transduction in cells from normal and hypereosinophilic subjects.. J Leukoc Biol.

[41] Yu W, Bozza PT, Tzizik DM, Gray JP, Cassara J (1998). Co-compartmentalization of MAP kinases and cytosolic phospholipase A2 at cytoplasmic arachidonate-rich lipid bodies.. Am J Pathol.

[42] Bandeira-Melo C, Weller PF, Bozza PT (2011). EicosaCell - an immunofluorescent-based assay to localize newly synthesized eicosanoid lipid mediators at intracellular sites.. Methods Mol Biol.

[43] Bandeira-Melo C, Phoofolo M, Weller PF (2001). Extranuclear lipid bodies, elicited by CCR3-mediated signaling pathways, are the sites of chemokine-enhanced leukotriene C4 production in eosinophils and basophils.. J Biol Chem.

[44] Vieira-de-Abreu A, Assis EF, Gomes GS, Castro-Faria-Neto HC, Weller PF (2005). Allergic challenge-elicited lipid bodies compartmentalize in vivo leukotriene C4 synthesis within eosinophils.. Am J Respir Cell Mol Biol.

[45] Pacheco P, Vieira-de-Abreu A, Gomes RN, Barbosa-Lima G, Wermelinger LB (2007). Monocyte chemoattractant protein-1/CC chemokine ligand 2 controls microtubule-driven biogenesis and leukotriene B4-synthesizing function of macrophage lipid bodies elicited by innate immune response.. J Immunol.

[46] Rodriguez-Acosta A, Finol HJ, Pulido-Mendez M, Marquez A, Andrade G (1998). Liver ultrastructural pathology in mice infected with Plasmodium berghei.. J Submicrosc Cytol Pathol.

[47] Pulido-Mendez M, Finol HJ, Giron ME, Aguilar I (2006). Ultrastructural pathological changes in mice kidney caused by Plasmodium berghei infection.. J Submicrosc Cytol Pathol.

[48] Pinheiro RO, Nunes MP, Pinheiro CS, D'Avila H, Bozza PT (2009). Induction of autophagy correlates with increased parasite load of Leishmania amazonensis in BALB/c but not C57BL/6 macrophages.. Microbes Infect.

[49] Cardona PJ, Llatjos R, Gordillo S, Diaz J, Ojanguren I (2000). Evolution of granulomas in lungs of mice infected aerogenically with Mycobacterium tuberculosis.. Scand J Immunol.

[50] Tanigawa K, Suzuki K, Nakamura K, Akama T, Kawashima A (2008). Expression of adipose differentiation-related protein (ADRP) and perilipin in macrophages infected with Mycobacterium leprae.. FEMS Microbiol Lett.

[51] Rank RG, Whittimore J, Bowlin AK, Wyrick PB (2011). In vivo ultrastructural analysis of the intimate relationship between polymorphonuclear leukocytes and the chlamydial developmental cycle.. Infect Immun.

[52] Pacheco P, Bozza FA, Gomes RN, Bozza M, Weller PF (2002). Lipopolysaccharide-induced leukocyte lipid body formation in vivo: innate immunity elicited intracellular Loci involved in eicosanoid metabolism.. J Immunol.

[53] Mattos KA, Oliveira VG, D'Avila H, Rodrigues LS, Pinheiro RO (2011). TLR6-driven lipid droplets in Mycobacterium leprae-infected Schwann cells: immunoinflammatory platforms associated with bacterial persistence.. J Immunol.

[54] McLauchlan J (2009). Hepatitis C virus: viral proteins on the move.. Biochem Soc Trans.

[55] Dvorak AM, Dvorak HF, Peters SP, Shulman ES, MacGlashan DW (1983). Lipid bodies: cytoplasmic organelles important to arachidonate metabolism in macrophages and mast cells.. J Immunol.

[56] Kumar Y, Cocchiaro J, Valdivia RH (2006). The obligate intracellular pathogen Chlamydia trachomatis targets host lipid droplets.. Curr Biol.

[57] van Manen HJ, Kraan YM, Roos D, Otto C (2005). Single-cell Raman and fluorescence microscopy reveal the association of lipid bodies with phagosomes in leukocytes.. Proc Natl Acad Sci U S A.

[58] Bougneres L, Helft J, Tiwari S, Vargas P, Chang BH (2009). A role for lipid bodies in the cross-presentation of phagocytosed antigens by MHC class I in dendritic cells.. Immunity.

[59] Yla-Anttila P, Vihinen H, Jokitalo E, Eskelinen EL (2009). 3D tomography reveals connections between the phagophore and endoplasmic reticulum.. Autophagy.

[60] Pandey AK, Sassetti CM (2008). Mycobacterial persistence requires the utilization of host cholesterol.. Proc Natl Acad Sci U S A.

[61] Luo M, Fadeev EA, Groves JT (2005). Mycobactin-mediated iron acquisition within macrophages.. Nat Chem Biol.

[62] Renz H, Gong JH, Schmidt A, Nain M, Gemsa D (1988). Release of tumor necrosis factor-alpha from macrophages. Enhancement and suppression are dose-dependently regulated by prostaglandin E2 and cyclic nucleotides.. J Immunol.

[63] Freire-de-Lima CG, Nascimento DO, Soares MB, Bozza PT, Castro-Faria-Neto HC (2000). Uptake of apoptotic cells drives the growth of a pathogenic trypanosome in macrophages.. Nature.

[64] Steinberg BE, Grinstein S (2008). Pathogen destruction versus intracellular survival: the role of lipids as phagosomal fate determinants.. J Clin Invest.

[65] Anes E, Kuhnel MP, Bos E, Moniz-Pereira J, Habermann A (2003). Selected lipids activate phagosome actin assembly and maturation resulting in killing of pathogenic mycobacteria.. Nat Cell Biol.

[66] Suh CI, Stull ND, Li XJ, Tian W, Price MO (2006). The phosphoinositide-binding protein p40phox activates the NADPH oxidase during FcgammaIIA receptor-induced phagocytosis.. J Exp Med.

[67] Liu P, Ying Y, Zhao Y, Mundy DI, Zhu M (2004). Chinese hamster ovary K2 cell lipid droplets appear to be metabolic organelles involved in membrane traffic.. J Biol Chem.

[68] Fabrino DL, Leon LL, Genestra M, Parreira GG, Melo RCN (2011). Rat models to investigate host macrophage defense against Trypanosoma cruzi.. J Innate Immun.

[69] Melo RCN, Machado CRS (1998). Depletion of radiosensitive leukocytes exacerbates the heart sympathetic denervation and parasitism in experimental Chagas' disease in rats.. J Neuroimmunol.

[70] Nicolaou G, Goodall AH, Erridge C (2012). Diverse bacteria promote macrophage foam cell formation via Toll-like receptor-dependent lipid body biosynthesis.. J Atheroscler Thromb.

[71] Qadri F, Bhuiyan TR, Dutta KK, Raqib R, Alam MS (2004). Acute dehydrating disease caused by Vibrio cholerae serogroups O1 and O139 induce increases in innate cells and inflammatory mediators at the mucosal surface of the gut.. Gut.

[72] Paraje MG, Correa SG, Renna MS, Theumer M, Sotomayor CE (2008). Candida albicans-secreted lipase induces injury and steatosis in immune and parenchymal cells.. Can J Microbiol.

